# First report of thyroid goiter in the marine ornamental fish *Gramma brasiliensis*

**DOI:** 10.29374/2527-2179.bjvm003624

**Published:** 2024-08-15

**Authors:** Sérgio Leandro Araújo-Silva, Maria Alcina Martins de Castro, Ricardo Evandro Mendes, Giovana Pelisser, Vanessa Martins da Rocha, José Victor Safadi Ferrarezi, Renata Ávila Ozório, Mônica Yumi Tsuzuki

**Affiliations:** 1 Biologist, DSc., Laboratório de Peixes e Ornamentais Marinhos (LAPOM), Departamento de Aquicultura, Centro de Ciências Agrárias, Universidade Federal de Santa Catarina (UFSC), Florianópolis, SC, Brazil.; 2 Veterinarian, DSc., Departamento de Aquicultura, UFSC, Florianópolis, SC, Brazil.; 3 Veterinarian, DSc., Athens Veterinary Diagnostic Laboratory (AVDL), Department of Pathology, College of Veterinary Medicine, University of Georgia (UGA), Athens, GA, United States of America.; 4 Veterinarian, Laboratório de Patologia Veterinária, Instituto Federal Catarinense (IFC), Concórdia, SC, Brazil.; 5 Fisheries engineer, DSc., Laboratório de Piscicultura Marinha (LAPMAR), Departamento de Aquicultura, Centro de Ciências Agrárias, UFSC, Florianópolis, SC, Brazil.; 6 Aquaculture engineer, Departamento de Aquicultura, UFSC, Florianópolis, SC, Brazil.; 7 Aquaculture engineer, DSc., LAPOM, Departamento de Aquicultura, Centro de Ciências Agrárias, UFSC, Florianópolis, SC, Brazil.; 8 Oceanographer, DSc., LAPOM, Departamento de Aquicultura, Centro de Ciências Agrárias, UFSC, Florianópolis, SC, Brazil.

**Keywords:** aquaculture, captive-breeding, iodine, nitrate, aquicultura, criação em cativeiro, iodo, nitrato

## Abstract

Enlargement of the thyroid gland is referred to as goiter. In captive fish, goiter may be associated with iodine deficiency in water or diet, exposure to goitrogenic factors such as a high environmental nitrate concentration or water treatment with ozone. This report describes the occurrence of goiter in a marine ornamental fish raised in a research laboratory, the Brazilian basslet *Gramma brasiliensis.* From 2016 to 2023, we observed progressively growing tumour-like masses in the pharyngeal cavity and along the gill arches of approximately 20 adult individuals. This abnormal growth impaired the ingestion of food and caused dyspnoea, leading the animals to death within a few months after the first appearance of the mass. The samples were submitted to histological analyses, which revealed moderate to severe, diffuse, hypertrophy and hyperplasia of thyroid follicular cells with most lacking colloids. This is the first report of goiter in the Brazilian basslet. Although it is not clear why this condition develops in this species, we recommend keeping nitrate levels to a minimum and monitoring water iodine concentrations regularly until future studies investigate the possible causes and adequate treatment for this species.

## Resumo

O aumento da glândula tireóide é conhecido como bócio. Em peixes em cativeiro, o bócio pode estar associado à deficiência de iodo na água ou na dieta, à exposição a fatores goitrogênicos, como alta concentração de nitrato ou ao tratamento da água com ozônio. O presente relato descreve a ocorrência de bócio em um peixe ornamental marinho criado em laboratório de pesquisa, o *Gramma brasiliensis*. De 2016 a 2023, observamos crescimento progressivo de massas semelhantes a tumores na cavidade faríngea e ao longo dos arcos branquiais de aproximadamente 20 indivíduos adultos. Esse crescimento anormal prejudicou a ingestão de alimentos e causou dispneia, levando os animais à morte em poucos meses, desde o início do aparecimento das massas. As amostras foram submetidas à análise histológica, evidenciando hipertrofia e hiperplasia moderada a grave, difusa, de células foliculares tireoidianas, sendo a maioria delas desprovida de colóide. Este é o primeiro relato de bócio em grama brasileiro. Apesar da razão pouco clara para o desenvolvimento desta condição nesta espécie, recomendamos manter os níveis de nitrato ao mínimo e monitorar regularmente as concentrações de iodo na água até que próximos estudos investiguem as possíveis causas e o tratamento adequado para esta espécie.

## Introduction

Enlargement of the thyroid gland is referred to as goiter and has been observed in different species of wild and mostly captive elasmobranchs (Crow et al., 2001; Gridelli et al., 2003; Morris et al., 2011), as well as captive marine teleosts (Jalenques et al., 2020; Ribeiro et al., 2011). Goiter has also been reported in freshwater fish such as zebrafish raised in research laboratories (Murray et al., 2018). Goiter is a result of hyperplasia or hypertrophy of the thyroid follicles with different morphological alterations, including follicular epithelial hyperplasia and/or alteration in colloid accumulation ranging from an absence to an abundant presence of colloids (Gridelli et al., 2003).

In captive fish, goiter may be associated with iodine deficiency in water or diet (Jalenques et al., 2020; Murray et al., 2018), since fish obtain iodide from both their diet via the gastrointestinal tract and from water via the gills (Brown et al., 2004), exposure to goitrogenic factors such as a high environmental nitrate concentration (Morris et al., 2011), and water treated with ozone (Morris et al., 2012). In the first case, iodine deficiency compromises the normal production of thyroxine and increases the production of thyroid-stimulating hormone (TSH) (Murray et al., 2018). Elevated TSH causes a hyperplasic response characterized by increased thickening of the thyroid follicular epithelium and follicle proliferation (Nishioka et al., 1987).

This report describes the occurrence of goiter in a marine ornamental fish raised in a research laboratory, the Brazilian basslet *G. brasiliensis* Sazima, Gasparini & Moura, 1998, which is a species endemic to the Brazilian coast, distributed from the state of Maranhão (including the Parcel de Manoel Luís) to Rio de Janeiro, and also found in the archipelagos of Fernando de Noronha (Sazima et al., 1998) and Abrolhos (Ferreira & Maida, 2006). This species is of interest to the ornamental trade which has affected its natural stocks, resulting in its inclusion on Brazil’s endangered species list in 2004 (Brasil, 2004). Its capture, transport and sales are currently prohibited throughout Brazil, except captive-bred individuals from aquaculture enterprises (Brasil, 2021). Thus, any study related to this species’ health conditions is of interest to the ornamental aquarium trade.

## Case report

**Figure 1:**
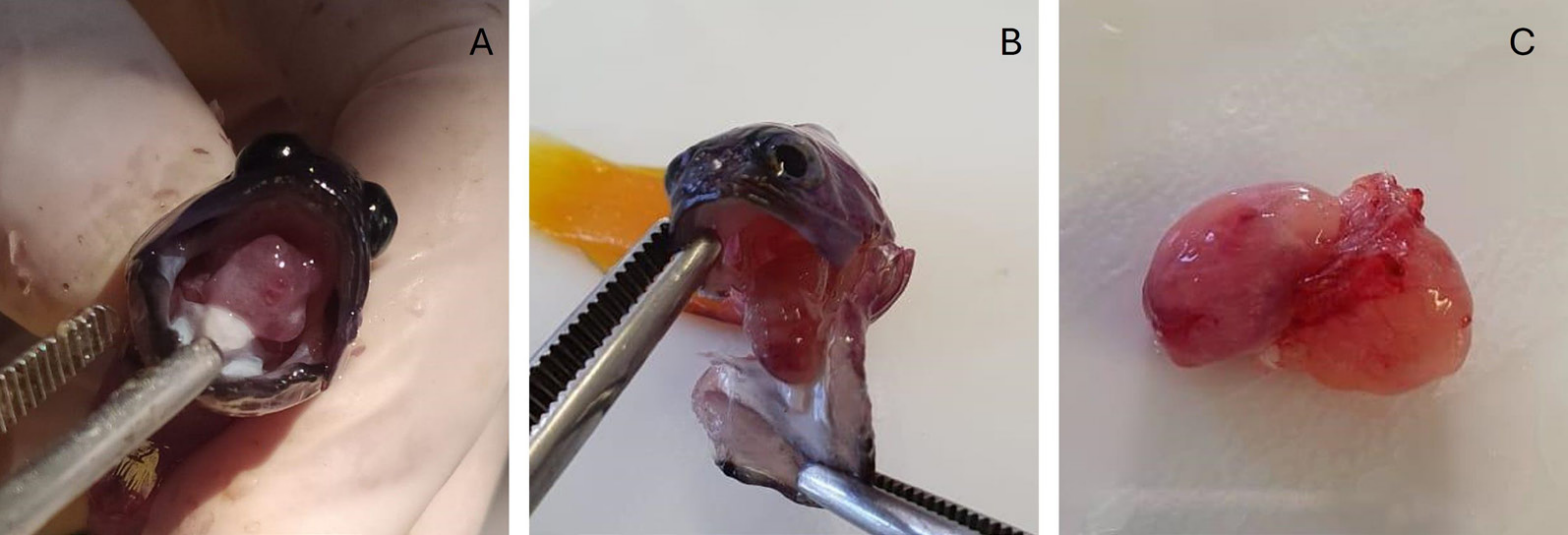
(A) Frontal view showing a pharyngeal nodule from a 7.1 cm adult *Gramma brasiliensis* partially to totally occluding the pharyngeal passage; (B) Lateral view showing the reach of the nodule towards the pharyngeal passage; (C) Excision of nodule showing bilateral enlargement of thyroid gland.

The Laboratory of Marine Fish and Ornamentals (LAPOM), Federal University of Santa Catarina (UFSC), Brazil, has been conducting research on *G. brasiliensis* reproductive biology and aquaculture since 2016. During these years, we observed progressively growing tumour-like masses (Figure 1) in the pharyngeal cavity and along the gill arches of approximately 20 adult individuals (7.1 to 9.2 cm total length). This abnormal growth impaired the ingestion of food and caused dyspnoea, leading the animals to death in a few months after the masses appeared.

Interestingly, no other species kept in the Laboratory (e.g. clownfish *Amphiprion ocellaris*, orchid dottyback *Pseudochromis fridmani*, barber goby *Elacatinus figaro*, flameback angelfish *Centropyge aurantonotus*, and the seahorse *Hippocampus reidi*) presented this abnormality, even though they shared the same seawater supply and similar housing systems (some clownfish shared the same system with *G. brasiliensis*).

Fish were kept in pairs, in individual glass aquaria with volumes of 70 to 110 L equipped with air stones, with PVC pipes placed to serve as shelters and nest spots. The aquaria were connected to water recirculation systems with natural seawater (salinity 34 to 36 ppt), photoperiods of 12 h light/12 h dark and temperature from 25 to 29 °C. Parameters such as pH (7.8 to 8.2) (YSI EcoSense pH 10, Yellow Springs, OH, USA), total ammonia and nitrite (<0.25 ppm) (LabconTest Marine, Alcon®) were measured weekly. The nitrate measurements (Red Sea test kit, Red sea®) were performed every two months, varying from 5 to 20 ppm. An ICP (Inductively Coupled Plasma) spectroscopy test of the seawater (taken from the Moçambique beach - the same location that supplies water to the laboratory) was carried out in 2021 and showed iodine levels of 58 µg L^-1^. All systems had a sump composed of a biological substrate, a mechanical filter, and a protein skimmer, and were syphoned daily, followed by a 5 to 10% water change.

Broodstock feeding was performed twice daily (08:30 and 16:00hours) until apparent satiety. In the mornings, a paste prepared in the laboratory composed of fresh marine shrimp (40%), fish (salmon 15%, sardines 15%), squid (10%), bivalve molluscs (mussels 10%, oysters 5%, scallops 5%) and a vitamin premix (1 g/kg) mixed in a blender, was provided. The afternoon feeding was comprised of a varied diet that included commercial marine ornamental fish feed (38% crude protein and 7% crude fat; Tropical, Chorzów, Poland), metanauplii and adult *Artemia* sp. (enriched with the microalgae *Chaetoceros muelleri* and *Isochrysis galbana*) and frozen marine shrimp postlarvae (*Litopenaeus vannamei*) obtained from local producers.

To find the cause of these growing masses, fish with severe conditions (i.e., with very large masses and not eating) were euthanized and had their masses removed (n=10 individuals). The samples were submitted to histological analyses. Fragments measuring 1cm thick were collected, fixed in 10% buffered formalin (Merk KGaA – catalog number R03379-86) for 48 hours, and dehydrated in ethyl-alcohol solutions (Sigma-Aldrich – catalog number R8382-1GA) of increasing concentrations (70-80-90-100%) during one hour in each, embedded in paraffin wax (78 °C per 3 hours), and then cut in sections ranging from 3 to 4 μm thick. The tissue sections were stained with haematoxylin-eosin (HE) according to Cardiff et al. (2014). This study was performed with the authorisation of the Ethics and Animal Use Committee of the Federal University of Santa Catarina (Number 3189250520).

Approximately 10 fish were found dead over these years and were not submitted to histological analyses, due to their advanced autolysis stage.

**Figure 2:**
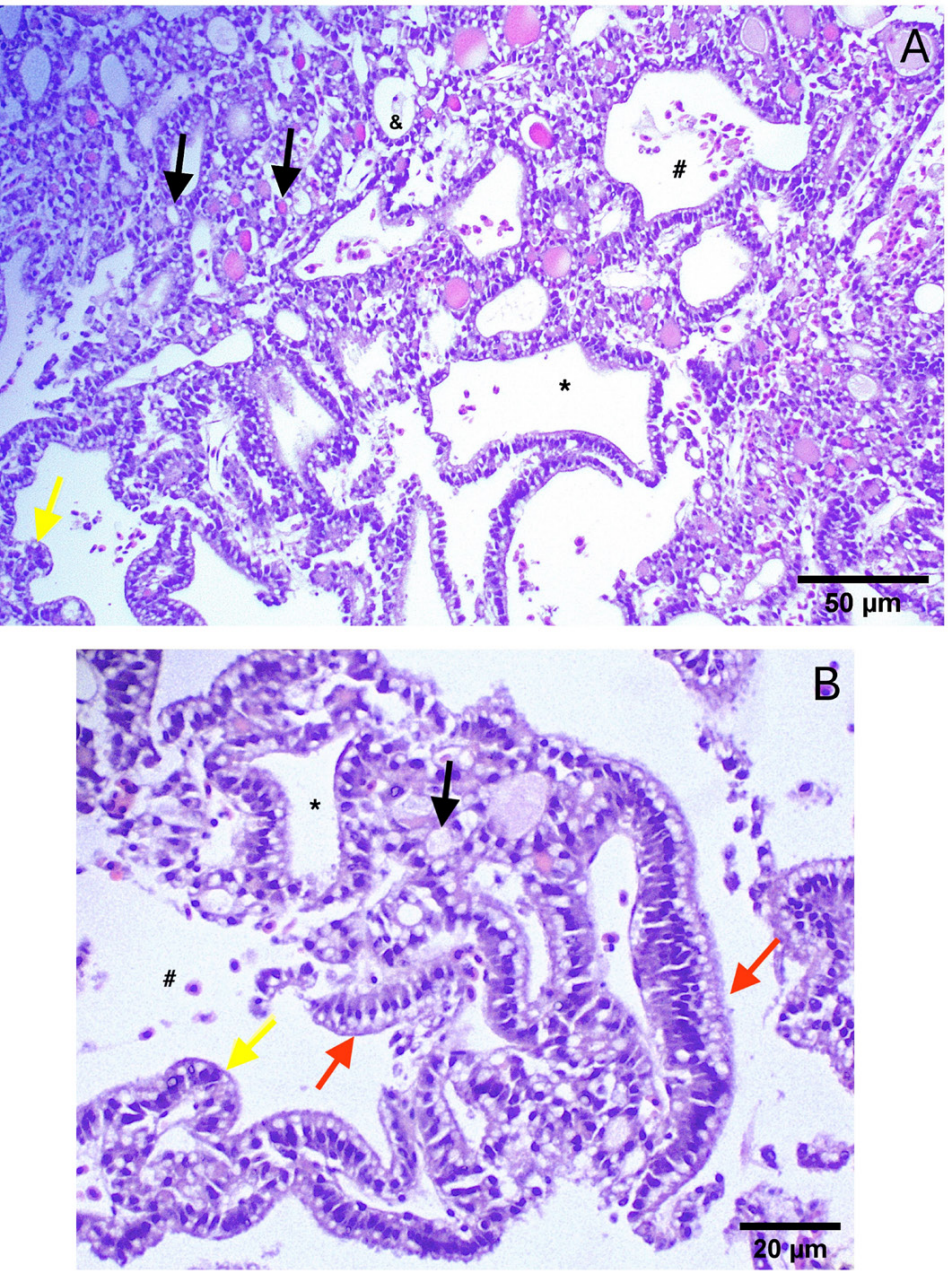
Thyroid gland, hyperplastic goiter. (A) Follicles in general lack colloids (*) and are dilated. A few follicles present a small amount of pale eosinophilic colloid, with reduced luminal diameter or no lumina (black arrow). Follicles have fused to form a large pseudo lumen containing blood and debris (#). A few follicles present a cuboidal epithelium (&); (B) Follicular epithelium is mainly cylindric, sometimes stratified, hyperplastic and vacuolated (red arrow). Marked papillary infolding of the follicular epithelium lining the fused follicle (yellow arrow) can be seen in large, fused follicles. A few follicles present a small amount of pale eosinophilic colloid, with reduced luminal diameter (black arrow). H&E stain.

Histopathological analyses showed moderate to severe, diffuse, hypertrophy and hyperplasia of thyroid follicular cells with most lacking colloids (Figure 2). A marked increase in the number of follicles was observed, varying moderately in size and shape. Some large follicles presented abundant colloids and others presented small acidophilic droplets of colloids, up to 3 micrometres in diameter. There were also several collapsed follicles, with a small amount of colloids or lacking them completely. Cells lining the follicles were cuboidal and larger follicles present two or three layers of follicular cells. The lesion was classified as a nodular follicular cell hyperplasia, according to Fournie et al. (2005). No evidence of cellular atypia, anisokaryosis or mitotic activity (average of two mitoses in 2.37 mm^2^ [equivalent to 10 FN22/40x fields]) were observed.

## Discussion

As *Gramma brasiliensis* is a marine fish, and seawater naturally contains iodine, when the masses first appeared we did not suspect goiter. After the diagnosis, there were no live animals with this condition, thus, a treatment such as supplementing their feed with iodine could not be performed and we did not investigate the iodine content in the recirculation system water.

However, the ICP test from the seawater in 2021 showed iodine levels of 58 µg L^-1^, and the iodine concentration in ocean waters is about 40-60 µg L^-1^ (Ito & Hirokawa, 2009; Shaw & Cooper, 1957; Tsunogai & Henmi, 1971). Even though the iodine concentration (IO_3_^-^ and I^-^) may decrease in a recirculation system due to consumption by plants and animals (Crow et al., 1998), we believe this consumption was irrelevant, since only fish were kept in the systems and there were daily water changes.

An occurrence of goiter induced by iodine deficiency in water was reported by Jalenques et al. (2020). They described the onset of goiter in dozens of ornamental marine fish kept in an aquarium after changing the brand of salt to produce artificial seawater. This salt contained less iodine. After switching back to salt with a higher iodine concentration, the authors reported that thyroid hyperplasia was no longer detected in any of the species.

An example of successful treatment via gastrointestinal tract is described by Gridelli et al. (2003). They observed regression of goiters in the elasmobranchs *Scyliorhinus canicular* and *S. stellaris* treated orally with potassium iodide (15 mg/kg). According to the authors, a progressive regression was observed after a month, with a reduction of clinical and behavioural signs.

Goiter can also be caused by iodide (I^−^) interaction with goitrogenic compounds (e.g., nitrate [NO_3_-N]) that inhibit the uptake of I^−^ into the thyroid gland (Crow et al., 2001). The recommended safe nitrate level for an aquarium shark exhibit is 70 mg/L or lower (Mohan & Aiken, 2004). Morris et al. (2011) related that an acute 29-d exposure to a nitrate concentration of 70 mg/L led to diffuse hyperplastic goiter in bamboo sharks. In this study, the nitrate did not exceed 20 mg/L. Although nitrate testing was sporadic, we believe it did not exceed this concentration, since the water was always changed regularly, and fish density was low (one pair per aquarium). It should be noted that we did not use ozone in the recirculation system.

## Conclusions

Although proliferative thyroid lesions are described in fish in the scientific literature, to our knowledge, this is the first report of goiter in the Brazilian basslet, *Gramma brasiliensis*. Although the reason for the development of this condition in this species is not clear, we recommend keeping nitrate levels to a minimum and regularly monitoring water iodine concentrations. Future studies should investigate the possible causes and the adequate treatment of thyroid goiter in this species. We also suggest that producers or hobbyists dose potassium iodide in food or water, according to recommendations in the literature for other species, while more studies regarding this condition on *G. brasiliensis* are performed.
